# Granular Calcite Stimulates Natural Mycorrhization and Growth of White Spruce Seedlings in Peat-Based Substrates in Forest Nursery

**DOI:** 10.3390/microorganisms8071088

**Published:** 2020-07-21

**Authors:** Mohammed S. Lamhamedi, Mario Renaud, Isabelle Auger, J. André Fortin

**Affiliations:** 1 Direction de la Recherche Forestière, 2700, rue Einstein, Québec, QC G1P 3W8, Canada; mariorenaud@hotmail.com (M.R.); isabelle.auger@mffp.gouv.qc.ca (I.A.); 2 Centre d’étude de la Forêt, Faculté de Foresterie, de Géographie et de Géomatique, Université Laval, Pavillon Abitibi Price, Rue de la Terrasse, Québec, QC G1V 0A6, Canada; j.andre.fortin@videotron.ca

**Keywords:** ectomycorrhizal fungi, growth, mineral nutrition, peat-based substrate, physicochemistry, *Picea glauca*, forest nursery

## Abstract

The acidity of peat-based substrates used in forest nurseries limits seedling mineral nutrition and growth as well as the activity of microorganisms. To our knowledge, no study has yet evaluated the use of granular calcite as a covering material to increase pH, calcium and CO_2_ concentrations in the rhizosphere and ectomycorrhizal development. The objective is to compare different covering treatments on early colonization of the roots by ectomycorrhizal fungi, as well as the growth and calcium nutrition of white spruce seedlings in the forest nursery. Three treatments were used to cover the plant cavities (Silica (29 g/cavity; control treatment), Calcite (24 g/cavity) and calcite+ (31 g/cavity)) and were distributed randomly inside each of the five complete blocks of the experimental design. The results show that calcite stimulates natural mycorrhization. Seedlings grown with calcite have significant gains for several growth and physiological variables, and that the periphery of their root plugs are more colonized by the extramatrical phase of ectomycorrhizal fungi, thus improving root-plug cohesion. The authors discuss the operational scope of the results in relation to the tolerance of seedlings to environmental stress and the improvement of their quality, both in the nursery and in reforestation sites.

## 1. Introduction

The production of boreal forest seedlings in peat-based substrates presents a significant challenge because of the acidity and variations in the physical properties of these substrates over a production cycle. These factors limit oxygen availability and affect the absorption kinetics of mineral nutrients, microorganism activity, along with shoot and root growth [1,2,3,4]. The container cavities in which the seedlings are produced are usually covered with silica. Due to their chemical composition, the granules or fine particles of silica (SiO_2_) do not induce variations in the pH of the substrate. In order to increase the pH of acidic peat-based substrates, certain forest nurseries in Quebec (Canada) and elsewhere have used the addition of calcic (CaCO_3_) or dolomite (CaMg (CO_3_)_2_) lime powder to their substrates. Increasing the pH aims at improving the availability and absorption of mineral nutrients and seedling growth in forest nurseries [1,3,5,6,7]. However, the pH generated by lime addition is neither stable nor optimal for the mineral nutrition and growth of seedlings [8,9]. Rapid, uncontrolled pH increases of the peat-based substrate (up to values above 7) results in symptoms of mineral deficiency (e.g., iron, boron) and stunted coniferous and deciduous seedlings, which fail to meet morphophysiological quality standards [3,5,8].

Our previous work on the physical properties of peat-based substrates used for the production of white spruce (*Picea glauca* (Moench) Voss) seedlings in forest nurseries focused on optimizing texture, aeration, rhizosphere gas diffusion, fertility and water retention capacity [10,11]. Other work has led to the improvement of plant growth and root-plug cohesion for several forest species through the optimization of different growth techniques at the operational scale: container characteristics, water stress, short-day treatments [12,13], management of cumulative light intensity in relation to the optical characteristics of the tunnel and according to the growth stage [14], irrigation [15,16,17] and fertilization [18,19,20].

To further improve seedling growth and overall performance in nurseries and reforestation sites, special attention has been paid to the development of nursery seedling inoculation techniques using ectomycorrhizal spores and inoculum (liquid and solid) of selected strains or genotypes obtained by controlled cross-breeding between compatible monokaryons of ectomycorrhizal fungi [21,22,23,24]. The main challenges of nursery seedling inoculation were the optimization of substrate fertilization and physicochemical parameters, and the use of effective strains capable of rapidly colonizing roots and competing with natural strains. Laboratory work has previously been carried out to optimize pH for fungal growth in agar media in the absence [25] and presence of the host plant, in peat-based substrates, and in hydroponic systems [26,27,28,29]. However, to our knowledge, no studies have yet assessed the possibility of using granular calcite as covering material to improve rhizosphere chemistry. This calcite overlay aims at increasing pH, Ca and CO_2_ concentrations in order to stimulate early root colonization by ectomycorrhizal fungi naturally present in the nursery, extension of their extramatrical phase, and growth of seedlings in forest nurseries. In reality, when fine calcite particles are exposed to water (irrigation, rain, etc.), they will dissociate according to the following chemical reaction (1):

(1) $$\mathrm{Ca}{\mathrm{CO}}_{3}+H_{2}O\to {\mathrm{Ca}}^{2+}+{\mathrm{CO}}_{2}+2{\left(\mathrm{OH}\right)}^{-}$$

To improve seedling morphophysiological quality and to reduce the costs associated with artificial ectomycorrhizal inoculation while promoting natural root ectomycorrhization, our approach is to replace silica, currently used as covering material, by granular calcite. Our hypothesis is that granular calcite improves substrate pH, early root colonization by ectomycorrhizal fungi in response to increased concentrations of calcium and CO_2_ in the rhizosphere released by calcite, and seedling mineral nutrition and growth when compared to silica-based covering material. Unlike strains selected for artificial inoculation (which include only one or a few genotypes), naturally present ectomycorrhizal fungi are genetically diverse, adapted to different environmental stresses and compatible with forest species and the climatic conditions of the boreal forest [22,30].

In Quebec’s forest nurseries, the growing season is very short. Seedlings are fertilized throughout the active growing season in order for them to reach the established morphophysiological quality standards (height, foliar nitrogen concentration, etc.). In this context, it is desirable to promote natural mycorrhization during the fall through ectomycorrhizal “spore showers” from forest stands located near these nurseries [22]. Improved morphophysiological quality and increased seedling ectomycorrhization will contribute to their survival and improve their tolerance to environmental stresses once they are planted at reforestation sites [23,31]. Indeed, with climate change in the boreal forest, transplanted forest seedlings will be subjected to more severe and frequent environmental stresses (drought, frost, pathogens, etc.).

Thus, the main objectives of this study are: (i) to compare the effects of granular calcite and silica on the main physicochemical characteristics of the substrate, on the early extension of the extramatrical phase of ectomycorrhizal fungi and on the growth and calcium nutrition of containerized white spruce seedlings during the second growing season (2+0) in a nursery, and (ii) to compare logistic models of seedling growth based on covering material (silica, calcite) and the presence or absence of ectomycorrhizal colonization. 

## 2. Materials and Methods

### 2.1. Plant Material, Covering Material and Experiment Design

White spruce seeds (seedlot: EPB-V3-EST-2-0; production code GP35EPB14-P85) were sown in 25−10 containers (model IPL, Saint-Damien, QC, Canada; 25 cavities, 310 cm^3^/cavity; 206 seedlings/m^2^). The containers were placed in a tunnel on 7 May 2014. The cavities were filled with a mixture of peat-based substrate and vermiculite (80%:20%, *v*/*v*), and bulk density was adjusted to 0.10 g/cm^3^. The pH_water_ of the initial substrate prior to seeding was 3.4. In the substrate, large fibers (retained on 2 mm sieves), large and medium fibers (greater than 0.850 mm), short (retained on 0.075 mm sieves) and fine fibers (those not retained on 0.075 mm sieves) accounted for 24.5%, 43.3%, 55.4% and 1.3% of the growing medium, respectively.

After the seeding operation, the seeds were covered with one of three types of materials, corresponding to the treatments: Silica (29 g/cavity; control treatment), Calcite (24 g/cavity) and Calcite+ (31 g/cavity). The amount of silica used in this project corresponds to the amount currently used at the operational scale. Calcite quantities, on the other hand, were established based on the results of our previous work [32], taking into account cavity volume and the effect of calcite on substrate pH. After potting and seeding, precise quantities of the covering material were placed over each container by an automatic spreader. The silica and calcite used were of high physicochemical quality and had no adverse effect on root and shoot growth.

All three treatments (silica, calcite and calcite+) were randomly distributed within each of the five complete blocks of the experimental design. Each treatment consisted of 27 containers (25–310cc) per block, for a total of 10,125 seedlings throughout the entire experimental design (excluding buffer zones). To eliminate border effects, buffer zones (27 containers/zone) were added between treatments and between blocks.

The containers were placed in one of the production tunnels of the Grandes-Piles governmental forest nursery (latitude: 46°43′54″ N; longitude: 72°42′06″ W). During the first growing season, the tunnel was covered with a 4 mm-thick milk-white polyethylene, with an incident light diminishing factor of 50–55% (Ginegar Plastic Products Ltd., multi-layer greenhouse cover film, type UVA/white 45%). The cover was retractable on both sides of the tunnel to increase ventilation and control the air temperature inside. The cover was removed around 6 October 2014, so the plants were exposed to outdoor conditions during their first winter and second growing season.

Germination was evaluated three times per week (Monday, Wednesday and Friday) in 60 containers (4 containers × 5 blocks × 3 treatments) during the first 31 days after seeding. This assessment showed that the mean cavity occupancy rate for all 3 treatments was 99.33%. Once germination was complete, the germinants were thinned to one per cavity. No germinant was transplanted to empty cavities.

### 2.2. White Spruce Seedling Production Techniques

The seedlings were produced using the standard nursery cultural techniques used in Quebec to produce white spruce seedlings in containers [23,31].

Irrigation and fertilization were carried out using a robot (Aquaboom model, Industrie Harnois, Saint-Thomas-de-Joliette, QC, Canada) to eliminate the effects of spatial variability in the substrate’s water content [15]. The robot’s coefficient of uniformity varied from 95% to 98%. Substrate water content was adjusted for each seedling growth stage during the first season (1+0) [16] and maintained between 40% and 45% (*v*/*v*) during the second season (2+0) [15]. Substrate water content (%, *v*/*v*) was monitored by gravimetry prior to irrigation [13].

The fertilization regime was adjusted every two weeks during both seasons (1+0 and 2+0) using the PLANTEC software, according to the growth stages [19]. By the end of the first growing season, each plant received 47.3 mg of nitrogen (N: 25.0 mg N-NH_4_, 22.3 mg N-NO_3_), 10.6 mg of phosphorus (P), 15.2 mg of potassium (K) and 0.1 mg of magnesium (Mg), as well as micronutrients (manganese, copper, iron and boron). During the second growing season (30 April 2015, to 9 October 2015), each plant received 250.3 mg of N (99.4 mg N-NH_4_, 111.8 mg N-NO_3_, 39.1 mg N-Urea), 41 mg of P, 83 mg of K and 0.6 mg of Mg, in addition to trace elements. To prevent the seedlings from reaching undesirable height, as required per morphophysiological quality standards, 2+0 seedlings were not fertilized between 25 June and 13 August 2015.

### 2.3. Silica and Calcite Particle Size

To characterize the statistical distribution of grain size, an analysis of silica and calcite particle size was performed by sieving [33]. Silica and calcite were sampled in the nursery prior to potting from the bulk piles of each type of covering material. To do so, three 500 g composite samples were used for each type of covering material (silica or calcite).

### 2.4. Substrate Physicochemistry and Seedling Calcium Nutrition and Growth

During the second growing season (2+0), the physicochemical variables associated with substrate fertility and seedling morphophysiological variables (growth, mineral nutrition) were determined by seven destructive samplings conducted every three weeks from 25 May to 28 September 2015. Mineral nutrient analyses of seedlings and substrate were conducted at the Laboratoire de chimie organique et inorganique (organic and inorganic chemistry laboratory) of the Direction de la recherche forestière (Quebec forest research branch), using the methods described in Lamhammedi et al. 2013, among others [18,34].

During each sampling, one container per treatment was randomly selected from each experimental block. In each of these containers, 15 of the 25 seedlings were randomly selected, for a total of 225 seedlings per sampling date (or 75 seedlings/treatment). The position of the container and the 15 sampled cavities were randomly selected. The same container and cavity positions were used for all block-treatment combinations at a given date.

Physicochemical characteristics and substrate fertility were assessed on one composite sample made of the 15 substrate root plugs per block and per treatment (root plugs were harvested along with the seedlings sampled for growth and mineral nutrition measurements). Substrate physicochemical variables include pH_water_, pH_CaCl_2__, electrical conductivity, and the mineral concentration and content of mineral nitrogen, phosphorus, potassium, calcium, and magnesium. In addition to pH_water_, which is commonly used by forest nurseries, pH_CaCl_2__ was also measured to better characterize and approximate the real variations taking place in the physicochemistry of the mycorrhizosphere after calcite hydrolysis [34].

Several morphophysiological variables were measured on the sampled seedlings: shoot height and diameter (15 seedlings/block/treatment), shoot and root dry masses (3 composite samples of 5 seedlings/block/treatment), and mineral nutrition of seedlings (1 composite sample of 15 seedlings/block/treatment). The root system was first cleaned by a system with a jet of compressed air. The roots were then washed to remove any particles of peat. Roots were placed in paper pouches before drying and mineral nutrient analysis.

Root and shoot calcium concentrations were analyzed using a plasma atomic emission spectrometer (model: ICAP 9000, Thermo Instruments, Franklin, MA, USA). Calcium content was calculated by multiplying the concentration by the dry mass to reflect the amount of calcium contained in a given amount of plant material [35].

### 2.5. Superficial Ectomycorrhizal Colonization of Seedling Root Plugs

Natural mycorrhization of seedling roots was assessed once at the end of the second growing season. In Quebec, white spruce is produced under a tunnel during the first growing season and transferred outdoors for the second year. The tunnel acts as a barrier to spore showers and that is why ectomycorrhizal colonization is almost non-existent at stage 1+0. At the end of the second growing season (27 October 2014) of the first growing season under tunnel conditions, we examined root tips of 225 white spruce seedlings (75 seedlings/treatment, 15 seedlings/treatment1/block) and no mycorrhiza formation was observed. 

At the end of the second growing season (2+0), we visually assessed the percentage of superficial growth of the ectomycorrhizal fungi’s extraradical mycelium (extramatrical phase) on the outside of each root plug. To do this, we randomly sampled 20 seedlings per container for each treatment in each block, for a total of 300 seedlings.

### 2.6. Statistical Analysis and Modeling of Growth Variables

#### 2.6.1. Analysis of Variance

To assess the effects of the cover treatment and the sampling date on substrate fertility variables and seedling morphophysiological variables (growth, mineral nutrition, etc.) measured during the second growing season, variance analyses were performed using mixed linear models with the MIXED procedure of SAS/STAT version 14.1 (SAS Institute Inc. 2015. Cary, NC, USA) using the average of samples per block/treatment. Treatment, sampling date, and their interaction were considered fixed effects, while the block and the interaction between the block and treatment was considered to be random. A model with a fixed effect (treatment) and a random block effect was used to analyze the percentage of ectomycorrhizal colonization. In all models, the number of degrees of freedom of the denominator for the fixed effects tests was calculated using the Satterthwaite method. The number of degrees of freedom at the denominator is therefore specific to each variable analyzed.

When the fixed effect was significant at the 5% threshold, orthogonal contrasts were performed. The treatment effect was broken down into two contrasts: mean calcite/calcite+ vs. Silica, on the one hand, and calcite vs. calcite+, on the other hand. If the interaction between date and treatment was significant, treatment contrasts were performed for each date. The effect of time was broken down into two contrasts: linear and quadratic. When the interaction between treatment and date was significant, the temporal evolution was compared between the treatments using these two contrasts. Thus, the linear effect and the quadratic effect of time were compared for calcite/calcite+ vs. silica treatments, and for calcite vs. calcite+ treatments. The contrasts tests were based on a simulation method with the ADJUST = SIMULATE option in SAS/STAT (version 14.1).

The hypotheses of variance normality and homogeneity were checked graphically. Where necessary, a residual variance by date was estimated to take into account variance heterogeneity. Preliminary analyses showed that sampling dates were not associated to a temporal correlation between the data.

Pearson correlation coefficients were calculated between the substrate’s different physicochemical variables.

#### 2.6.2. Modeling of Growth Variables

The temporal evolution of each of the growth variables (*y*) was modeled with the logistic function (2) using a mixed nonlinear model and the NLMIXED procedure of SAS/STAT version 14.1 (SAS Institute Inc.):

(2) $$y=\frac{a+u}{1+e^{-c\left(day-b\right)}}$$

where parameters *a*, *b*, and *c* are, respectively, the asymptote, inflection point and growth rate. Parameter *u* is the random effect of the interaction between the block and the treatment.

To test for differences in the kinetics of the *assessed* growth variables between the three treatments during the growing season, indicator variables were added to the model (3). These variables are defined as *i*_1_ = 1 if (treatment = calcite, otherwise, *i*_1_ = 0, and *i*_2_ = 1 if treatment = calcite+, otherwise, *i*_2_ = 0.

(3) $$y=\frac{a_{0}+a_{1}i_{1}+a_{2}i_{2}+u}{1+e^{-\left(c_{0}+c_{1}i_{1}+c_{2}i_{2}\right)\left(day-\left(b_{0}+b_{1}i_{1}+b_{2}i_{2}\right)\right)}}$$

when there was no significant difference between calcite and calcite+ for a given parameter, only one parameter common to both treatments was used (*a*_12_, *b*_12_, *c*_12_). The model was then simplified by removing the indicator variables that were not significant at the 5% threshold. Models were adjusted to the dataset for the different sampling dates.

Residue normality and homogeneity of variance hypotheses were checked graphically. To account for the heterogeneity of the variances, the residual variance was weighted according to the variance observed at each date. Logistic model adjustment was checked graphically by comparing the predicted values with the averages observed at each time per treatment. A coefficient of determination (R^2^) (4) was calculated as follows:

(4) $$R^{2}=1-\left[\sum {\left(y-\hat{y}\right)}^{2}/\sum {\left(y-\bar{y}\right)}^{2}\right]$$

where $\hat{y}$ is the value predicted by the model and is $\bar{y}$ the mean of *y*.

## 3. Results

### 3.1. Silica and Granular Calcite Particle Size

**Figure 1:**
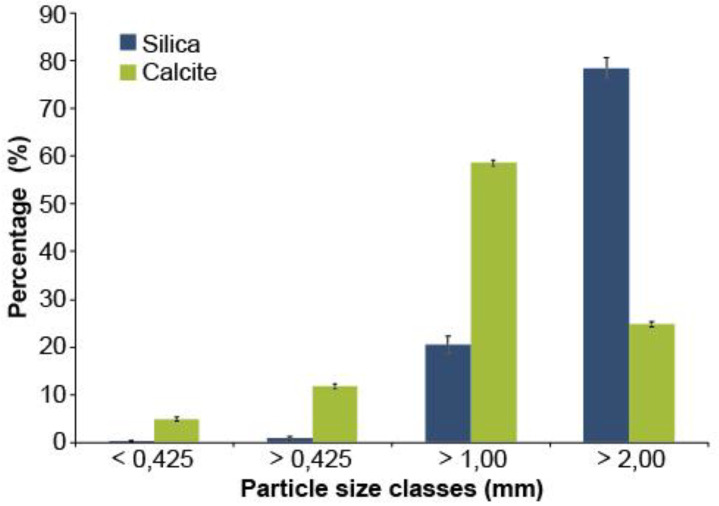
Particle size distribution of silica and calcite used as covering material during the production of white spruce seedlings (*n* = 3 composite samples of each covering material. The error bars represent the standard error).

The particle size analysis shows that silica and calcite differ in their particle size proportions (Figure 1), and that these two materials do not have the same distribution according to the four observed particle size classes (very fine: 0.425 mm; medium: >1.00 mm; coarse: >2.00 mm). Calcite contains a much larger proportion of very fine and fine particles (16.7%), and medium particles (58.6%) than silica (1.1% and 20.5%; Figure 1), while silica contains much more coarse particles (78.3%) than calcite (24.7%).

### 3.2. Physicochemical Properties and Fertility of the Substrate

The effects of the Date × Treatment interaction, the date (linear and quadratic effects), and the treatment were significant for most substrate physicochemical and fertility variables (pH_water_, pH_CaCl_2__, N_min_, P, K, Ca, and Mg) measured during the second growing season (2+0) (Table 1). In all cases, no significant difference was observed between the two calcite treatments (calcite vs. calcite+: *p* ≥ 0.5398). Except for conductivity, the mean of the two calcite treatments (calcite/calcite+) was significantly different from that of the silica treatment for all assessed substrate physicochemical and fertility variables.

**Figure 2:**
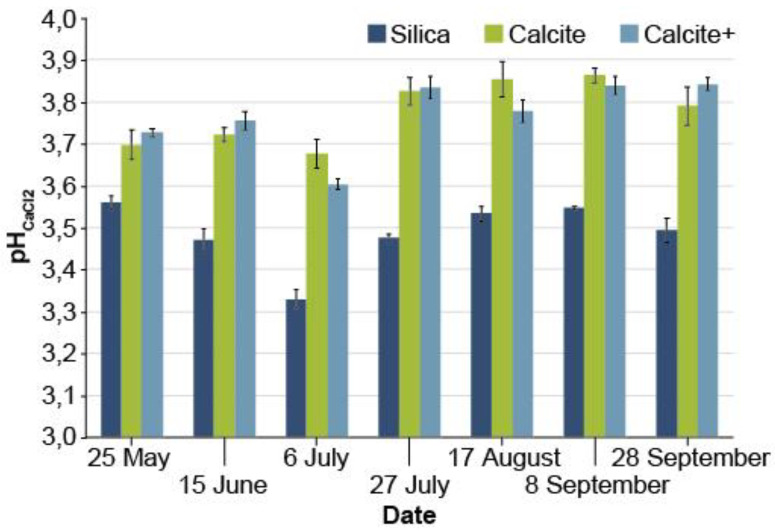
Evolution of pH_CaCl_2__ of peat growth substrate during the growing season of white spruce seedlings (2+0), depending on the covering material (silica: 29 g/cavity, calcite: 24 g/cavity and calcite+: 31 g/cavity).

Orthogonal contrasts show that mean substrate pH was significantly higher in calcite and calcite+ treatments than silica during the second growing season for both pH_CaCl_2__ (Table 1, Figure 2) and pH_water_ (Table 1). At the end of the second growing season (28 September 2015), both pH values were higher in calcite/calcite+ treatments (pH_water_ = 4.9 and pH_CaCl_2__ = 3.8) than in silica treatments (pH_water_ = 4.4 and pH_CaCl_2__ = 3.4).

The Ca concentration was significantly higher in calcite-covered substrates than in silica-covered substrates for all sampling dates. For example, at the last sampling date, the substrate’s Ca concentration was significantly greater in calcite/calcite+ treatments (125.5 ± 10 ppm) than in silica treatments (43 ± 13 ppm). Substrate pH and Ca concentrations were significantly correlated (pH_eau_: r = −0.60, pH_CaCl_2__: r = 0.30, *p* = 0.002).

### 3.3. Superficial Ectomycorrhizal Colonization of Seedling Root Plugs

**Figure 3:**
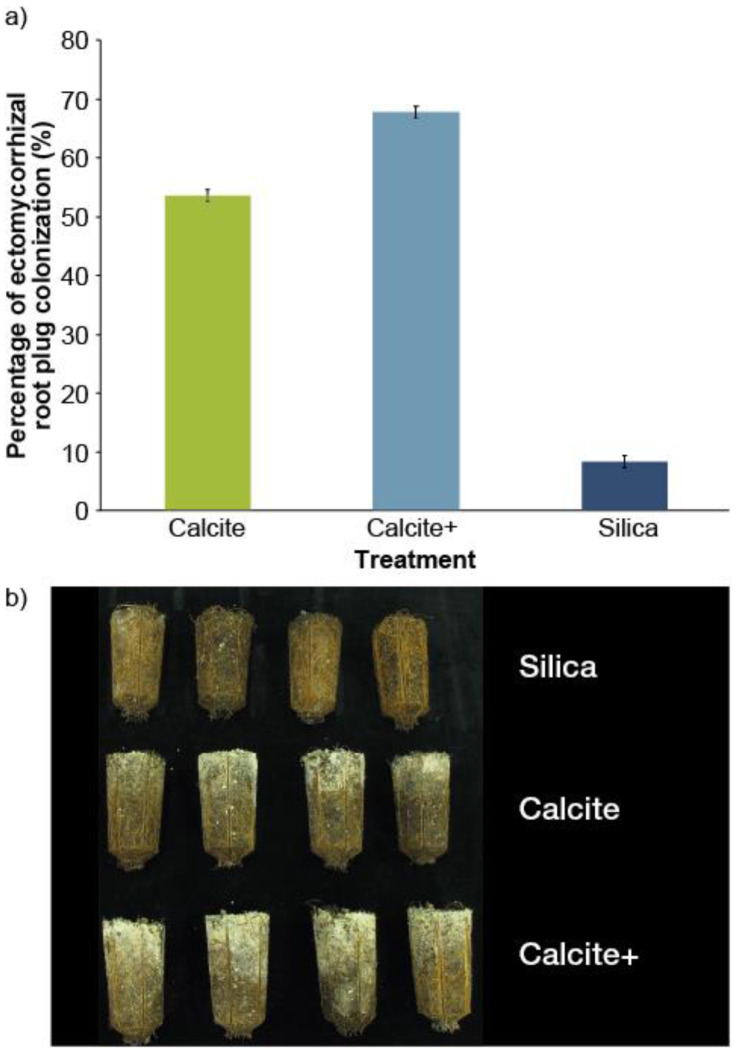
(**a**) Percentage of superficial colonization of the root system of white spruce (2+0) seedlings (n = 100 seedlings/treatment) by the extramatrical phase of the ectomycorrhizal fungi *Laccaria bicolor* in autumn (28 September, 2015); (**b**) example of variability in the surface colonization of root plugs of white spruce seedlings (2+0) by *L. bicolor* depending to the covering material (Silica: 29 g/cavity, Calcite: 24 g/cavity and Calcite +: 31 g/cavity, volume of each cavity = 310 cm^3^).

At the end of the second growing season, the percentage of superficial colonization of white spruce seedling root plugs by the extraradical mycelium (e.g., extramatrical phase) of the ectomycorrhizal fungus significantly varied with the covering material (*p* < 0.0001). Orthogonal contrasts showed that the mean degree of root plug surface colonization by ectomycorrhizal fungi was significantly greater (*p* < 0.0001) in calcite/calcite+ treatments (61%) than in silica treatments (8%) (Figure 3a,b). However, the difference was not significant (*p* = 0.1045) between calcite (54%) and calcite+ (68%) treatments. Colonization by the extramatrical phase of ectomycorrhizae showed the same pattern on all root plugs examined, i.e., from the top (covering material) to bottom (Figure 3b).

The majority of seedlings were colonized by *Laccaria bicolor* (Maire) P.D.Orton, the most abundant ectomycorrhizal fungus in Quebec forest nurseries. The dichotomous structures of ectomycorrhizae, of *L. bicolor* are easily visible to the naked eye on the surface of the root plugs of white spruce seedlings. On a microscopic scale, these structures are characterized by the presence of mantle hyphae and Hartig net hyphae. Fruiting bodies of this fungus are very abundant in association with several forest species (white spruce, black spruce (*Picea mariana* [Mill.] B.S.P.), jack pine (*Pinus banksiana* Lamb.), white pine (*Pinus strobus* L.), etc.). *L. bicolor* can easily be identified by its rubber smell, lilac-colored base, white spore-print, and subglobose to ellipsoid echinulate spores (Rolland Labbé, Mycoquébec). Of the 300 sampled seedlings, only one had been superficially colonized by *Telephora terrestris* Ehrhenberg Pl. Crypt. Linn. Exsicc. on a small portion (˂25%) of the root plug area.

### 3.4. White Spruce Seedlings Growth during the Second Growing Season (2+0)

The Date × Treatment interaction was significant (*p* ≤ 0.0433) for all growth variables except root diameter and dry mass (Table 2). Simple effects of treatment and date were significant (*p* ≤ 0.0005) for all growth variables.

The mean diameter of the seedlings that received the calcite treatment was not significantly different from that of the seedlings who received the calcite+ treatment (*p* = 0.2906). However, the combined mean diameter of the seedlings who received calcite and calcite treatments was significantly greater than that of the silica treatment (*p* = 0.0001; Table 2). Mean root dry mass differed significantly between calcite and calcite+ treatments (*p* = 0.0293) and between calcite/calcite+ and silica treatments (*p* = 0.0007).

The differences between calcite and calcite+ treatments were not significant at any of the sampling dates for the other four growth variables (height (H), height to diameter ratio (H/D), shoot mass, and total dry mass). At most of these dates, the average of calcite/calcite+ treatments was nonetheless significantly higher than that of silica treatments.

**Figure 4:**
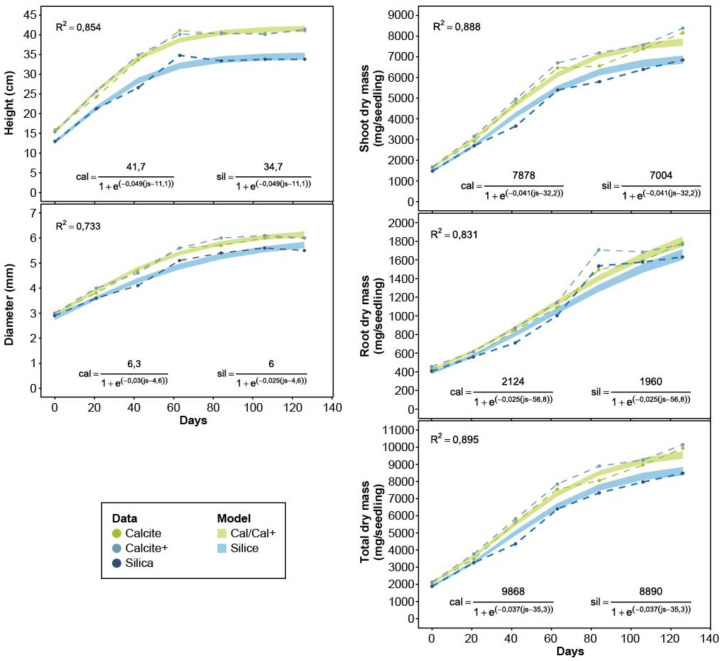
Evolution of the means and logistic models adjusted for each of the growth variables of white spruce seedlings (2+0), depending on the treatments (silica, calcite and calcite+). The width of each band corresponds to the 95% confidence interval. As the parameters of the calcite and calcite treatments did not differ significantly from each other, a single logistic model was generated by combining the data from these two treatments. The models were adjusted using all the data (n = 525 seedlings/treatment for height and diameter, and n = 105 composite samples of 5 plants/treatment for shoot, roots and total dry masses).

At the end of the second growing season, seedlings treated with calcite and calcite+ showed significant increases in height (21.7% on average), diameter (9.1% on average), shoot dry mass (20.6%), and total dry mass (18.3%) compared to silica treatments (Figure 4).

### 3.5. Logistic Growth Models

Kinetics of the different growth variables are well adjusted to the logistic models (R^2^ ranging from 0.733 to 0.895, Figure 4). Many of these model parameters significantly differ between the silica treatment and the two calcite treatments (calcite/calcite+) (Figure 4, Table S1), but those of the calcite and calcite+ treatment models do not differ significantly from one another. This shows that white spruce (2+0) seedlings that had their cavities covered with calcite have significant height, diameter, and dry mass gains (for the shoot and root, and for the whole plant) (Figure 4). For example, the asymptote of mean seedling height at the end of the second growing season was 41.7 cm in calcite/calcite+ treatments, but only 34.7 cm in the silica treatment.

### 3.6. Seedling Calcium Nutrition

The Date × Treatment interaction was significant for the calcium content (*p* < 0.0001), but not for the calcium concentration (*p* = 0.0742) in shoots. This interaction was also significant for root calcium content (*p* < 0.0001) and concentration (*p* = 0.0124). Simple effects of treatment and sampling date were significant for calcium content and concentration in the shoot and roots.

**Figure 5:**
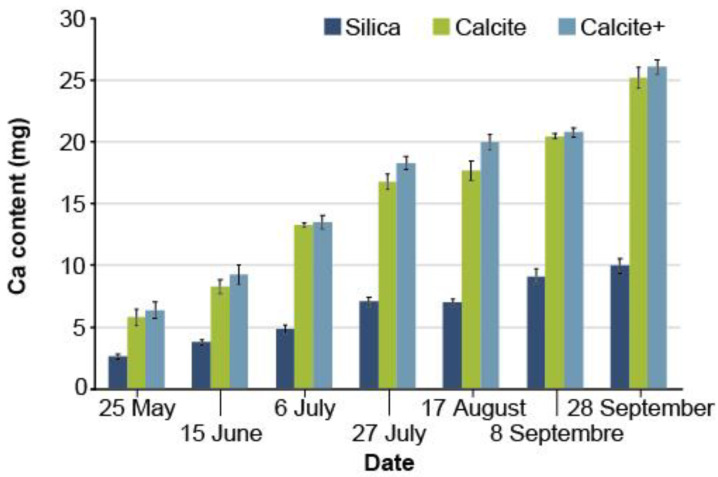
Evolution of the average mineral content of the shoots of white spruce seedlings (2+0) in calcium (Ca), depending on the treatments (silica, calcite and calcite +) (*n* = 5 composite samples of 15 plants each. The error bars represent the standard error).

Orthogonal contrasts of the shoot calcium content showed no significant difference between calcite and calcite+ treatments regardless of the sampling date (*p* > 0.0530). Even so, the mean shoot calcium content was significantly higher (*p* < 0.0001) in the combined calcite/calcite+ treatments than in the silica treatment. The difference was significant at all sampling dates and it even increased during the growing season (Figure 5). 

With respect to the calcium concentration and content in the roots, the significant superiority of calcite/calcite+ treatments over the silica treatment persisted throughout the growing season, and at the last sampling date, the concentration (calcite/calcite+: 0.36 ± 0.01%; silica: 0.21 ± 0.01%) and Ca content (calcite/calcite+: 6.34 ± 0.20 mg; silica: 3.46 ± 0.26 mg) for the calcite/calcite+ treatments were almost double those of silica.

## 4. Discussion

The use of calcite rather than silica as covering material significantly improved the chemical properties of the peat-based substrate (pH and Ca concentration), calcium nutrition, and the different growth variables of white spruce seedlings (2+0) when produced at an operational scale. The use of calcite as covering material also significantly increased mycorrhization and surface colonization of root plugs (2+0) by extraradical mycelium of *L. bicolor*, which was not observed with silica. The development of extraradical mycelium helps improve root plug cohesion and decrease insufficient root development (Figure 3). 

### 4.1. Calcite: A Key Factor in Stimulating Mycorrhization and Growth of the Extramatrical Phase of Ectomycorrhizal Fungi

**Figure 6:**
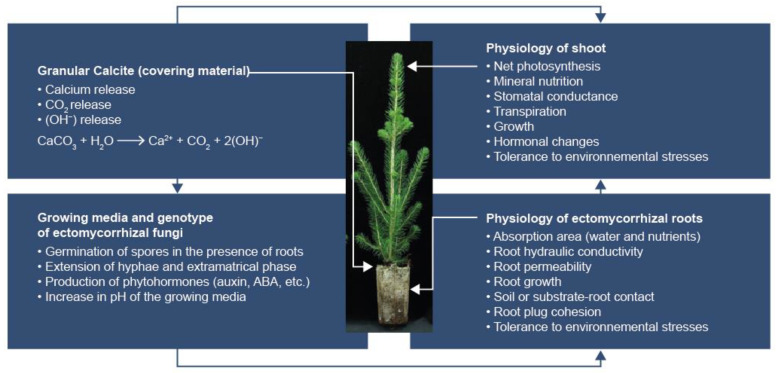
Main mechanisms by which calcite and ectomycorrhizae improve the physicochemistry of the substrate, and the growth and physiology of plants.

Early ectomycorrhizal colonization and rapid extension of extraradical mycelium outside root plugs of calcite-covered white spruce seedlings (2+0) (Figure 3) are synergically stimulated by several factors, including improved substrate pH, Ca and CO_2_ release in the rhizosphere by calcite (Figure 6) and probably by the presence of secondary microorganisms in the fungal mantle of ectomycorrhizae [36]. Indeed, it has been demonstrated that the inoculation of jack pine with a combination of *Laccaria bicolor* and *Pseudomonas fluorescens* significantly increases seedling growth relative to the effects of either of these treatments (fungus or bacterium) applied alone [37]. 

Granular calcite (CaCO_3_) used as covering material releases CO_2_ [38], which is combined with that generated in the rhizosphere by microorganism activity (including ectomycorrhizae) and root respiration [39,40]. This additional CO_2_ stimulates spore germination, and the growth and development of ectomycorrhizal fungal hyphae [41,42], biomass and fruiting bodies [43,44]. After spore germination, the release of Ca into the rhizosphere by calcite also stimulates the growth and extension of fungal hyphae [45]. This explains why the extramatrical phase of *L. bicolor* extends and grows rapidly with the increasing gradient of Ca concentration and pH (results not shown) from the top of the root plug (where the covering material was present) to the bottom (Figure 3b). It is highly likely that the germination of *L. bicolor* spores, in the presence of white spruce roots (2+0) [46], requires a minimum concentration of CO_2_, which is achieved faster in the presence of calcite than silica. Indeed, calcite has the high CO_2_ emission factor of 44% [38], while silica does not release CO_2_. This partly explains the fast growth of *L. bicolor*’s extramatrical hyphae in the presence of calcite. It has been shown that hyphal growth is optimal at a CO_2_ concentration of 2.5% for certain endomycorrhizal species (e.g., *Gigaspora margarita* Becker and Hall) [47]. 

Root architecture and root plug cohesion of white spruce seedlings were improved following ectomycorrhizal colonization (Figure 3). These observations are consistent with those described by several authors [22,48,49]. The anatomical characteristics of ectomycorrhizae and the very high density of extraradical mycelium in the extramatrical phase of *L. bicolor* significantly increase root length and facilitate the exploration of substrate volume. The extraradical mycelium of *L. bicolor* is directly connected to seedling roots and represents a functional extension of the root system. For example, the length of the ectomycorrhizal fungus’s hyphae under controlled conditions can be 10–80 m per cm of root [50]. Further work [51,52] has shown that the extension of the extraradical mycelium’s surface (hyphae and mycelial cords) of *Pisolithus* sp. Coker and Couch significantly improves root architecture and surface absorption by *Pinus pinaster* (Aits) seedlings in sandy soil. The measured extension of the extramatrical phase’s surface was 1–3 cm^2^/day depending on the fungus’s genotype.

The extramatrical development of ectomycorrhizal fungi also improves soil and substrate structure, particularly the arrangement and cohesion of aggregates by hyphae and extracellular polysaccharide secretions [53,54]. Read [55] estimates that for some ectomycorrhizal fungi, the length of the extramatrical hyphae can reach 200 m/g dry soil or 2000 m/cm^3^ of fresh soil and that the density of the hyphae in the active area of extension can reach 250 individual filaments per mm linear. 

The extramatrical phase of ectomycorrhizal fungi increases the absorption surface of plant roots (water and mineral elements). For white spruce seedlings produced in containers (350 cm^3^/cavity) in a peat-based substrate, the mean total length of all roots measured by image analysis (n = 36 plants) over the entire growing season (1+0) at an operational scale did not exceed 834 cm under optimal irrigation control (45%, *v*/*v*), i.e., 0.02 m/cm^3^ of fresh substrate [15]. The presence of these fine hyphae, small in diameter relative to the roots, allows the seedlings to explore the full volume of the substrate and to penetrate between the substrate particles where the roots cannot access. 

Mycorrhizal colonization also helps reduce the leaching of mineral elements by retaining and immobilizing these elements in its various structures (hyphae, fungal mantle, etc.) [56]. For example, a portion of Ca is absorbed on the hyphae’s surface in the form of calcium oxalate (CaOx) crystals [57]. The formation of CaOx was significantly increased by the presence of bicarbonates released by calcite and nitrates from fertilization [58]. In this regard, Arocena et al. [59] showed that when *Piloderma* is associated with the roots of *Picea glauca* (Moench) Voss x Picea engelmannii Parry, the surface of its hyphae is covered with Ca-rich inlays (e.g., CaOx) with a calcium concentration reaching up to 17% Ca. These hyphae store significant amounts of mineral elements and help reduce leaching and pollution of the water table. After the hyphae die, Ca is released into the soil solution and absorbed by the roots [57].

Calcite increases pH to an optimal level (pH_water_ = 4–5) for growth and mineral nutrition of boreal forest conifers produced in peat substrates [5], as well as for growth and development of ectomycorrhizal fungi from the boreal forest [25]. Other factors and interactions between pH and Ca also contribute to improved substrate physicochemistry, plant growth and physiology (Figure 6).

Our previous results and observations on the main boreal forest species (black spruce, white spruce, jack pine, and larch) produced in Quebec’s forest nurseries have shown that root colonization by the extramatrical phase of *L. bicolor* provides good root plug cohesion and helps reduce insufficient root development [22,48,49,60], which is one of the most important plant rejection criteria [14]. Thus, *L. bicolor* distinguishes itself by its extremely dense extramatrical phase (Figure 3b), which ensures good cohesion between substrate particles and roots [61]. Improved root plug cohesion by the dense extramatrical phase of *L. bicolor* has also been reported in white spruce cuttings (B+2) [49]. Maintaining this cohesion is essential for the qualification of nursery seedlings and in the different handling stages between the nursery and the planting site.

### 4.2. Calcite: An Asset for the Improvement of Growth and Morphophysiological Quality of Seedlings Produced in Forest Nurseries

The use of calcite rather than silica as covering material did not affect seed germination or cavity occupancy rate, which averaged 99.33% for all three treatments.

At the last sampling date of the second growing season, seedlings covered with calcite and calcite+ showed significant height gains over silica-covered seedlings (mean deviation of 21.7%), shoot dry mass (20.6%), and total mass (18.3%). Growth model asymptotes (Figure 4) clearly demonstrate the significant superiority of calcite over silica. For dry root mass, the 9% difference between calcite/calcite+ and silica treatments was not significant, perhaps because root growth was restricted by the cavities (310 cm^3^) during the second growing season. However, at the end of the first growing season and when cavity volume (root living space) was not a limiting factor, root mass was significantly greater with the calcite+ treatment than the silica treatment (31% difference; calcite+: 562.0 ± 15.0 mg; silica: 427.0 ± 12.0 mg; results not shown). 

In terms of mineral nutrition, the mean shoot Ca content in calcite-covered cavities was significantly higher than that of silica-covered plants (Figure 5). Calcium, alone or through synergy with other factors (ectomycorrhizae, CO_2_, etc.), significantly increased seedling growth due to its positive effects on different physiological processes. This element activates cell division and elongation, regulates cell membrane permeability, strengthens cell wall rigidity and acts as a regulator of photosynthesis in synergy with abscisic acid during stomatal opening [62,63,64]. Calcium also stimulates photosynthesis and nitrogen, phosphorus, and potassium absorption [65,66]. For other crops (e.g., tobacco), the addition of Ca improves the absorption of trace elements, namely iron, manganese, zinc and boron [67].

In recent years, nursery growers have experienced significant seedling losses due to extreme weather conditions characterized by delayed accumulation of cold hours in autumn and lack of snow cover in early winter [68]. To this end, the use of calcite as covering material along with improved natural seedling ectomycorrhizal inoculation could contribute to improved hardening and frost tolerance of seedlings. Indeed, studies have shown that hardening and frost tolerance are enhanced by calcium supplementation in forest plant nurseries [69] and in fruit and forest trees grown in the field such as apple trees, green oaks and red spruces [70,71,72]. The presence of ectomycorrhizae in Scots pine (*Pinus sylvestris* L.) has also been shown to increase hardening and frost tolerance [73,74]. In our view, the combination of calcium and ectomycorrhizal supplementation, the subject of this study, allows the simple effects already observed of these two factors to work in synergy to increase seedling tolerance to other environmental, abiotic (salinity, excess water, drought, high temperatures) [52,66,70,72,75]; and biotic stresses (attacks by pathogens) [76].

In Quebec, artificial inoculation by local *L. bicolor* spores yielded excellent results at an operational scale in terms of inoculation success and carpophore production. For example, a private nursery inoculated 1+0 and 2+0 seedlings of different forest species (jack pine, fir, white, black, red and Norway spruce) in seven tunnels to promote root plug cohesion and improve root growth [22,48]. This inoculation improved root plug cohesion and resulted in an *L. bicolor* carpophore density of 1–14.5 carpophores/m^2^, which represents an average of 61–744 carpophores per tunnel after evaluation of 210 to 600 containers per tunnel. The presence of ectomycorrhizal fungi also improves the plasticity and functional features of planted seedlings, including the acquisition, absorption and transport of water and mineral elements, gas exchanges, and tolerance to different environmental stresses [40,52,77,78].

## 5. Conclusions 

To our knowledge, this is the first assessment of the effects of granular calcite used as covering material on substrate physicochemistry, early root colonization by ectomycorrhizal fungi, growth and calcium nutrition of forest seedlings produced at an operational scale. 

Calcite significantly improved the physicochemistry of the mycorrhizosphere’s substrate, as well as the growth and mineral nutrition of nursery seedlings (Figure 6). The use of granular calcite instead of silica as covering material led to increased mycorrhization and development of the extramatrical phase of ectomycorrhizal fungi as a result of the interaction between three main factors (pH improvement, increase in Ca and CO_2_ concentrations). Increased root plug cohesion will reduce the number of plants discarded due to insufficient root development [48,49,60]. The number of plants discarded for other reasons may also decrease as the calcium released by calcite and ectomycorrhizal colonization increase the seedling’s tolerance to abiotic and biotic stresses (frost, pathogenic fungi, etc.).

The use of granular calcite will allow forest nursery workers to reduce fall applications of calcium nitrate (CaNO_3_), which is used as a fertilizer to provide the essential calcium needed for plant hardening and frost tolerance [20]. This decrease will also contribute to reducing nitrate leaching [15] and the cost of fertilizers.

This study led to the development of growth models for 2+0 white spruce seedlings grown using two types of covering material: silica and granular calcite. These models can be used as nursery stock standards by growers looking to optimize their growing techniques and increase the achievement rate of morphophysiological quality standards (during seedling delivery).

Our results on calcite and nursery ectomycorrhizae development, combined with those on the performance of seedlings inoculated with *L. bicolor* at reforestation sites during the juvenile phase [79,80,81,82,83], can contribute to the achievement of the objectives of the reforestation and assisted migration programs [79,80]. More particularly, by improving the survival rate and tolerance of seedlings to different environmental stresses during their establishment phase at reforestation sites.

Finally, calcite can also be used in agriculture, agroforestry and in horticultural nurseries to improve soil physicochemistry and the development of mycorrhizal fungi, as well as to improve yields and resistance to environmental stresses for different crops.

## Figures and Tables

**Table 1:** Observed probabilities (*p*-values) and degrees of freedom of the fixed effects associated with the analysis of the variance of the fertility and physicochemical variables of the substrate during the second (2+0) growing season of white spruce seedlings in a forest nursery. A *p*-value in bold indicates a significant Date × Treatment interaction at *p* < 0.05.

Source of Variation.	Degrees of Freedom *		*p* Values							
	DLN	DLD	pH_water_	pH_CaCl_2__	Cond ^†^ (µS/cm)	N_min_^†^ (mg/kg)	P ^†^ (mg/kg)	K ^†^ (mg/kg)	Ca ^†^ (mg/kg)	Mg ^†^ (mg/kg)
Treatment	2	8	<0.0001	0.0051	0.2459	0.0029	<0.0001	0.0012	<0.0001	0.0003
Calcite/calcite+ vs. silica	(1)	8	<0.0001	0.0017	0.0990	0.0008	<0.0001	0.0003	<0.0001	0.0001
Calcite vs. calcite+	(1)	8	0.6844	0.5398	0.9562	0.5734	0.9970	0.7603	0.5976	0.7471
Date	6	70	<0.0001	<0.0001	<0.0001	<0.0001	<0.0001	<0.0001	<0.0001	<0.0001
Date (linear effect)	(1)	70	<0.0001	<0.0001	<0.0001	<0.0001	<0.0001	<0.0001	<0.0001	<0.0001
Date (quadratic effect)	(1)	70	0.0213	<0.0001	<0.0001	<0.0001	<0.0001	<0.0001	<0.0001	<0.0001
Date × Treatment	12	70	<0.0001	0.0001	<0.0001	0.0001	<0.0001	<0.0001	<0.0001	<0.0001

* DLN: degrees of freedom of the numerator and DLD: degrees of freedom of the denominator according to Satterthwaite’s correction. The DLDs presented are those for pHeau and pHCaCl_2_. They vary from 13 to 30 for the other variables. ^†^ Cond.: electrical conductivity, N_min_: mineral nitrogen, P: phosphorus, K: potassium, Ca: calcium and Mg: magnesium.

**Table 2:** Observed probabilities (*p*-values) and degrees of freedom of the fixed effects associated with the analysis of the variance of the growth variables of white spruce (2+0) seedlings under forest nursery conditions. A value of p in bold indicates a significant effect at *p* < 0.05 of the Date × Treatment interaction, a Treatment effect or a Date effect.

Source of Variation	Degrees of Freedom *		*p* Values					
	DLN *	DLD *	Height (H, cm)	Diameter (D, mm)	H/D	Dry Mass		
						Shoots (mg)	Roots (mg)	Total (mg)
Treatment	2	12	<0.0001	0.0002	<0.0001	<0.0001	0.0005	<0.0001
Calcite/calcite+ vs. silica	(1)	12	<0.0001	0.0001	<0.0001	<0.0001	0.0007	<0.0001
Calcite vs. calcite+	(1)	12	0.6702	0.2906	0.3109	0.0408	0.0293	0.0310
Date	6	70	<0.0001	<0.0001	<0.0001	<0.0001	<0.0001	<0.0001
Date (linear effect)	(1)	71	<0.0001	<0.0001	<0.0001	<0.0001	<0.0001	<0.0001
Date (quadratic effect)	(1)	71	<0.0001	<0.0001	<0.0001	<0.0001	0.0192	<0.0001
Date × Treatment	12	70	<0.0001	0.0527	0.0433	0.0003	0.2998	0.0008

* DLN: degrees of freedom of the numerator and DLD: degrees of freedom of the denominator. The DLDs presented are those for height and diameter. They vary from 18 to 26 for shoot dry mass, 20 to 48 for root dry mass and 19 to 26 for total dry mass.

## Supplementary Materials

The following are available online at https://www.mdpi.com/2076-2607/8/7/1088/s1, Table S1: Estimation of the parameters of the logistic models of the evolution of the height, the diameter, the dry mass of the shoots, the roots and the total dry mass of the white spruce seedlings (2+0) in a forest nursery. The parameters in bold indicate that the difference between the Silica and (Calcite/calcite) treatments is significant.
