# Supplementary material for: Granular Calcite Stimulates Natural Mycorrhization and Growth of White Spruce Seedlings in Peat-Based Substrates in Forest Nursery
Source: Microorganisms. 2020 Jul 21;8(7):1088. doi: 10.3390/microorganisms8071088 (PMC7409261; doi:10.3390/microorganisms8071088)
Supplement: Supplementary file 1 [file microorganisms-08-01088-s001.pdf]

Table S1. Estimation of the parameters of the logistic models of the evolution of the height, the diameter, the dry mass of the shoots, the roots and the total dry mass of the white spruce seedlings (2+ 0) in a forest nursery. The parameters in bold indicate that the difference between the Silica and (Calcite/calcite) treatments is significant.

Variable	Parameter*	Estimated value	Error-type	p-value
Height (cm)	*a0*	34,7	0,4	< 0.0001
	a12	**7,0**	**0,5**	**< 0.0001**
	b	11,1	0,3	< 0.0001
	c	0,049	0,001	< 0.0001
Diameter (mm)	*a0*	6,0	0,1	< 0.0001
	a12	**0,3**	**0,1**	**0,0064**
	b	4,6	0,8	< 0.0001
	c0	0,025	0,002	< 0.0001
	c12	**0,005**	**0,002**	**0,0091**
Shoot dry mass (mg)	*a0*	7004	115	< 0.0001
	a12	**874**	**103**	**< 0.0001**
	b	32,2	1,0	< 0.0001
	c	0,041	0,001	< 0.0001
Root dry mass (mg)	*a0*	1960	64	< 0.0001
	a12	**164**	**32**	**< 0.0001**
	b	56,8	3,0	< 0.0001
	c	0,025	0,001	< 0.0001
Total dry mass (mg)	*a0*	8891	151	< 0.0001
	a12	**977**	**125**	**< 0.0001**
	b	35,3	1,2	< 0.0001
	c	0,037	0,001	< 0.0001

* Parameter *a* is the asymptote, parameter *b* is at the inflection point and parameter *c* is the rate of growth. Parameters *a0* and *c0* are associated with the silica treatment, while parameters *a12* and *c12* are associated with the treatments (calcite/calcite+); when the latter are different from zero (i.e. $p < 0.05$), the evolution of the treatment curve (calcite/calcite+) is significantly different from that of the silica treatment. Parameters *b* and *c* are common to all three treatments.
